# MitProNet: A Knowledgebase and Analysis Platform of Proteome, Interactome and Diseases for Mammalian Mitochondria

**DOI:** 10.1371/journal.pone.0111187

**Published:** 2014-10-27

**Authors:** Jiabin Wang, Jian Yang, Song Mao, Xiaoqiang Chai, Yuling Hu, Xugang Hou, Yiheng Tang, Cheng Bi, Xiao Li

**Affiliations:** College of Life Sciences, Sichuan University, Ministry of Education Key Laboratory for Bio-resource and Eco-environment, Sichuan Key Laboratory of Molecular Biology and Biotechnology, Chengdu, People’s Republic of China; Johannes-Gutenberg University of Mainz, Germany

## Abstract

Mitochondrion plays a central role in diverse biological processes in most eukaryotes, and its dysfunctions are critically involved in a large number of diseases and the aging process. A systematic identification of mitochondrial proteomes and characterization of functional linkages among mitochondrial proteins are fundamental in understanding the mechanisms underlying biological functions and human diseases associated with mitochondria. Here we present a database MitProNet which provides a comprehensive knowledgebase for mitochondrial proteome, interactome and human diseases. First an inventory of mammalian mitochondrial proteins was compiled by widely collecting proteomic datasets, and the proteins were classified by machine learning to achieve a high-confidence list of mitochondrial proteins. The current version of MitProNet covers 1124 high-confidence proteins, and the remainders were further classified as middle- or low-confidence. An organelle-specific network of functional linkages among mitochondrial proteins was then generated by integrating genomic features encoded by a wide range of datasets including genomic context, gene expression profiles, protein-protein interactions, functional similarity and metabolic pathways. The functional-linkage network should be a valuable resource for the study of biological functions of mitochondrial proteins and human mitochondrial diseases. Furthermore, we utilized the network to predict candidate genes for mitochondrial diseases using prioritization algorithms. All proteins, functional linkages and disease candidate genes in MitProNet were annotated according to the information collected from their original sources including GO, GEO, OMIM, KEGG, MIPS, HPRD and so on. MitProNet features a user-friendly graphic visualization interface to present functional analysis of linkage networks. As an up-to-date database and analysis platform, MitProNet should be particularly helpful in comprehensive studies of complicated biological mechanisms underlying mitochondrial functions and human mitochondrial diseases. MitProNet is freely accessible at http://bio.scu.edu.cn:8085/MitProNet.

## Introduction

Almost all eukaryotic organisms possess mitochondria as their essential cellular components that function as the center of energy production, metabolism, signaling, apoptosis and cell growth [1]. Mitochondrial dysfunctions are known to be associated with a broad spectrum of metabolic and age-related diseases in humans, including diabetes mellitus, several cancer types, cardiovascular disorders, and neurodegenerative diseases such as Alzheimer’s and Parkinson’s disease [2]–[6]. Since these mitochondria-related diseases are caused by multigenic factors and have complex clinical phenotypes, they still remain to be poorly understood and difficult to develop medical therapy. In mammals, it is estimated that the mitochondrion is composed of about 1500 distinct proteins, the vast majority of which (above 99%) are nuclear-encoded except for thirteen polypeptides of the respiratory chain that are encoded in the mitochondrial genome (mtDNA) [7], [8].

In order to understand better the roles mitochondria play in human health and disease, our priority is to define and characterize the mitochondrial proteome [9]. In the past few years, many research communities have made great efforts to identify mitochondrial proteins using different approaches, including genetics, proteomics and bioinformatics methods. In particular, mass spectrometry-based technologies exhibit the capability of high-throughput proteins identification, and have been widely utilized to define and characterize the mammalian mitochondrial proteome, which resulted in the publication of various proteomics data sets. Meanwhile, many web-accessible databases, such as MitoP2 [10], MitoProteome [11], MitoMiner [8], MitoRes [12], MiGenes [13] and MitoCarta [14], were developed to store the mitochondrial protein data that were curated manually from the biochemical literatures or collected from the large-scale proteomic studies. Among these, some performed the bioinformatics methods to improve the confidence and the coverage of mitochondrial proteomes [14].

Despite these significant successes in identifying mitochondrial proteins, the high complexity of the current data sets coupled with the tissue and development heterogeneity of mitochondrial proteins [15] are a major challenge to their use in understanding of the mammalian mitochondrial proteome and discovering susceptible genes in complex mitochondrial diseases. Firstly, a lack of common standards hinders us from defining the comprehensive and accurate mitochondrial proteome. By combining various experimental datasets from the proteomic studies, an integrative analysis showed that about 7300 proteins were identified as mitochondrial, which significantly excesses the estimated size of the mammalian mitochondrial proteome. The large number of proteins reveals the presence of false discovery in large-scale proteomic studies. This is mainly due to the purified mitochondria are often contaminated by other non-mitochondrial organelles such as microsomes and cytoskeletons whose proteins are falsely identified as mitochondrial [7]. Secondly, with the rapidly increasing number of newly discovered mitochondrial proteins, a critical task beyond protein identification is to annotate cellular functions for newly-identified mitochondrial proteins and to associate their functional roles with human mitochondrial disorders. The investigation [14] on MitoCarta which may represent the largest comprehensive compendium of mammalian mitochondrial proteins to date indicated that about a quarter of proteins in the inventory were not annotated to a biological process in terms of Gene Ontology (GO) annotation [16]. If we expand to the whole mitochondrial proteome, a greater number of mitochondrial proteins will remain to be uncharacterized.

With the increase in the availability of genomic and proteomic data, computational approaches have been proposed for inferring the biological function of mitochondrial proteins, prioritizing and predicting candidate genes susceptible to mitochondrial disorders. Many computational approaches follow the idea termed ‘guilt-by-association’ that the function of one protein could be transferred from another protein with known function relying on their biological relationship [17]. The large-scale genomic and proteomic datasets allow us to measure quantitatively the biological relationship between two genes, including gene expression profiling, protein-protein interactions, phylogenetic profiling, and synthetic genetic analysis and so on. For example, using phylogenetic profiling analysis across hundreds of species, Pagliarini *et al*. identified 19 novel factors that are involved in the assembly of complex I of the mitochondrial respiratory chain [14]. More recently, the biological relationships among a set of genes/proteins can be represented as a network such as gene co-expression network, transcription regulation network and protein interaction network, which provides us a global perspective of understanding mitochondrial biology and disease at a systems level [18]–[20]. Nevertheless, most of those studies on mitochondria used only individual data source or data type, which led to insufficient coverage of the mitochondrial proteome and thus potentially limited their predictive ability.

A reasonable alternative would be to utilize the functional linkage network (FLN) integrated from heterogeneous datasets generated from successful efforts on larger scale assembly. The integration of complementary knowledge from heterogeneous sources is essential to understand the system as a whole and obtain well populated networks. Comparing with the networks derived from individual data type, the FLNs are denser and less biased towards a kind of particular evidence. Many successes have been achieved in predicting gene functions and prioritizing disease genes through utilizing the FLN-based scheme. Although several FLN databases have been distributed, such as STRING [21], Reactome [22] and BioGRID [23], there are very few FLN databases that are designed specifically for mitochondria.

To address the issue of single data set or type, Franke et al. [24] constructed a functional linkage network (FLN) by integrating multiple types of genome-wide data, and utilized the FLN for disease gene prioritization. However, it is speculated that the performance of this FLN was highly dependent on Gene Ontology (GO) annotations, and as a result, the predictions tended to be biased towards well-characterized genes, and thus limit capacity on inferences. In another study, Linghu et al. [25] integrated multiple genome-wide features to construct an evidence-weighted FLN, and used a neighborhood-weighting decision rule for disease gene prioritization successfully. Nevertheless, while specialized in mitochondrion, a specific FLN among proteins in this organelle using a combination of multiple types of data focusing its message exclusively on functional associations among mitochondrial proteins, would deliver superior performance. To date, only two databases specialized for mitochondrial protein interactions are public available, Mitointeractome [26] and InterMitoBase [27]. Mitointeractome is a representative interaction database for mitochondria which includes predicted protein-protein interactions (PPIs) based on structural and homologous information. InterMitoBase contains well-annotated PPIs between mitochondrial and mitochondrial/non-mitochondrial proteins integrated from a wide range of resources. However, the both of databases cover only PPI information, which is not sufficient for characterizing functional associations among mitochondrial proteins. Therefore, it is necessary to construct a database covering the entire FLN that characterizes the global functional associations among mitochondrial proteins.

In this study, we performed a machine-learning classifier to integrate mitochondrial proteins from 23 proteomic datasets for compiling an inventory of mammalian mitochondrial proteins. Comparing with other datasets, the list of mitochondrial proteins comprising 1124 proteins reveals a larger coverage and better accuracy. A mitochondria-specific FLN was constructed by integrating 15 heterogeneous genomic and proteomic datasets, resulting in 32,951 weighted functional linkages among 1072 mitochondrial proteins. Furthermore, the mitochondria-specific FLN was utilized to identify and prioritize candidate genes for typical mitochondrial diseases. The results show the inventory of mitochondrial proteins and the FLN among mitochondrial proteins should be valuable resources in comprehensive studies of complicated biological mechanisms underlying mitochondrial functions and human mitochondrial diseases.

## Results and Discussion

### General procedure

**Figure 1:**
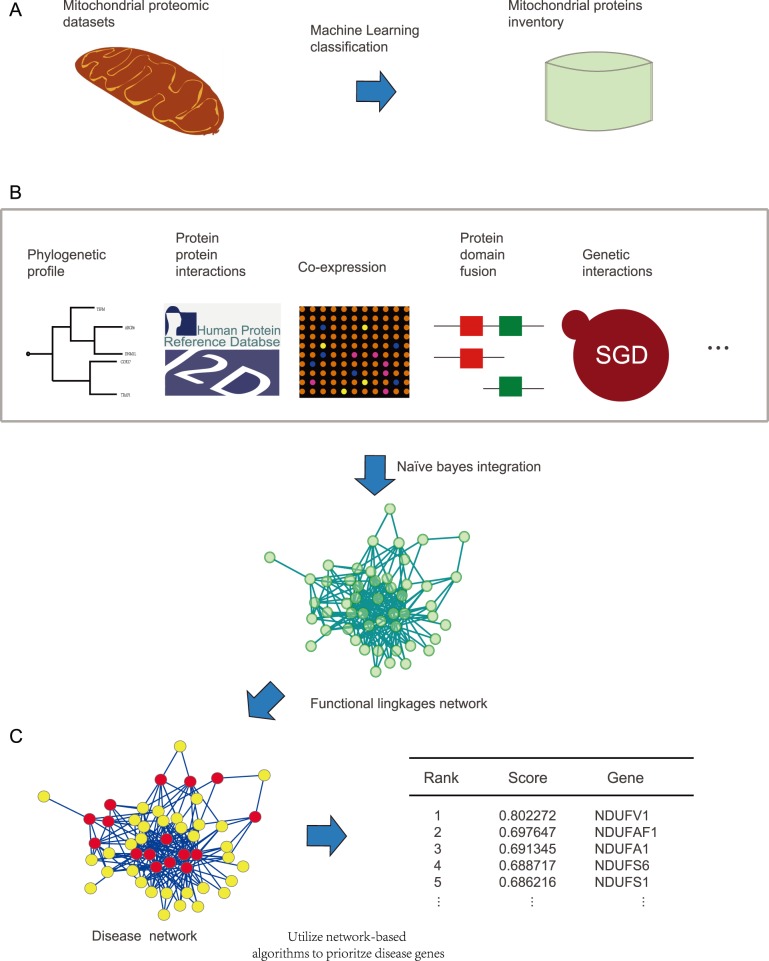
A flowchart depicting the work. (A) Step 1: obtaining a mitochondrial proteins inventory utilizing machine learning classification. (B) Step 2: constructing the FLN by integrating 11 genomic features including protein-protein interaction, domain-domain interaction, shared domains, genomic context, genetic interaction, phenotypic semantic similarity, co-expression, GO semantic similarity, protein expression profiles, disease involvement and operon based on the Naïve bayes model. (C) Step 3: ranking the disease candidate genes utilizing the FLN and a network-based algorithm. The table on the right shows the ranking scores of the top 5 candidate genes for mitochondrial complex I deficiency.

The overall procedure (Figure 1) included three steps. The first step was to compile an inventory of mammalian mitochondrial proteins by means of collection from various proteomic experimental datasets and several publicly-available databases. In the second step, a FLN among mitochondrial proteins was constructed through integrating functional features from heterogeneous ‘omic’ data sources. Finally, the FLN was then used to identify and prioritize candidate genes for mitochondrial diseases.

### An inventory of mammalian mitochondrial proteins

Although Pagliarini *et al*. presented the most comprehensive mammalian mitochondrial proteome (the MitoCarta database) with nearly 1100 proteins and estimated that their compendium covers more than 85% of the mitochondrial proteome [14], Meisinger *et al.* speculated that they may underestimate the size of the mammalian mitochondrial proteome and that the total number of mammalian genes for mitochondrial proteins could approach 1500 [28]. Considering the limitation of the databases, as a first step, we needed to compile an inventory of mammalian mitochondrial proteins that covers as many proteins as possible in the organelle. Thus, we made an extensive collection of mammalian mitochondrial proteins identified experimentally.

Despite various proteomics-scale experiments successfully identified mitochondrial proteins, a combined experimental datasets from these proteomic studies showed that about 7300 proteins were identified as mitochondrial proteins, which significantly exceeded the estimated size of the mammalian mitochondrial proteome. The large number of proteins reveals the presence of false discovery in large-scale proteomic studies. The previous investigation revealed that there is a high conservation among mammalian mitochondrial proteomes [8], hence it is a complement to compile a comprehensive inventory of mitochondrial proteins by integrating the proteomic datasets from a wide range of mammalian mitochondria. Here we collected 23 proteomic datasets from three model mammals including human (*H. sapiens*), mouse (*M. musculus*) and rat (*R. norvegicus*) for the integration (Table 1). To reduce false discovery, moreover, we performed a machine-learning classifier to integrate mitochondrial proteins.

**Table 1:** Integrated mitochondrial proteomic datasets for an inventory of mammalian mitochondrial proteins.

Datasets	Species	Number of Proteins	Tissue/organ/cell	Method
Calvo S et al. [29]	H. sapiens	1048		Prediction
Taylor SW et al. [30]	H. sapiens	600	Heart	MS
Rezaul K et al. [31]	H. sapiens	656	T leukemia cells	MS
Xie J et al. [32]	H. sapiens	180	Immortalized lymphoblastoid cell lines	2-GE
Ozawa T et al. [33]	M. musculus	48	Cell line BNL1ME (liver)	GFP
Mootha VK et al. [34]	M. musculus	462	Brain, heart, kidney, and liver	MS
Jin J et al. [35]	M. musculus	781	Dopaminergic cells	MS
Kislinger T et al. [36]	M. musculus	1872	Brain, heart, kidney, liver, lung, and placenta	MS
Da Cruz S et al. [37]	M. musculus	97	Liver	MS
Johnson DT et al. [15]	R. norvegicus	292	Brain, liver, heart, and kidney	MS
Forner F et al. [38]	R. norvegicus	503	Muscle, heart, and liver	MS
Reifschneider NH et al. [39]	R. norvegicus	110	Kidney, Liver, Heart, Skeletal Muscle and Brain	BN
Palmfeldt J et al. [40]	H. sapiens	2591	Skin fibroblast	MS
Lefort N et al. [41]	H. sapiens	892	Skeletal muscle	MS
Bousette N et al. [42]	M. musculus	2087	Heart	MS
Fang X et al. [43]	M. musculus	2165	Brain	MS
Zhang J et al. [44]	M. musculus	916	Heart	MS
Deng WJ et al. [45]	R. norvegicus	624	Liver	MS
Wu L et al. [46]	H. sapiens	1149	T leukemia cells	MS
Catherman AD et al. [47]	H.sapiens	1326	H1299 cells	MS
Hansen J et al. [48]	H.sapiens	2138	human lymphoblastoid cells	MS
Chappell NP et al. [49]	H.sapiens	1523	Epithelial ovarian cancer cell	MS
Chen X et al. [50]	R. norvegicus	1215	rat INS-1 cells	MS

MS, mass spectrometry. 2-GE, two-dimensional gel electrophoretic. GFP, green fluorescent protein. BN, blue-native.

We used weka, a software that collecting a set of machine learning algorithms for data mining tasks [51], to integrate mitochondrial proteomic datasets. As a first step of machine learning, a gold standard positive (GSP) set and gold standard negative (GSN) set were constructed. Based on the test set, various machine-learning classifiers including AdaBoostM1, Id3, J48, Logistic, MultiClassClassifier, MultilayerPerceptron, NaiveBayes and RandomForest were trained. We assessed the prediction performance by 10-fold cross-validation, showing that the AdaBoostM1 classifier [52] achieved the best, prediction with a high sensitivity of 0.93 (Table S1). The AdaBoost classifier was then applied to identify mitochondrial proteins form 23 proteomic datasets, which resulted in 1109 proteins as positives, 550 of which were the known mitochondrial proteins in the GSP set. There were 15 proteins defined in the GSP were falsely classified as non-mitochondrial proteins. To achieve a comprehensive database of mitochondrial proteins, the high-confidence list was curated manually to include these proteins. As a result, we created an inventory of high-confidence mammalian mitochondrial proteins that includes 1124 mitochondrial proteins (Table S2), which consists of 1109 proteins predicted by the AdaBoostM1 classifier as well as 15 missing proteins from the GSP set. In order to utilize sufficiently the proteomic resources, we further classified the remaining about 6100 proteins as middle-confidence or low-confidence using a simple voting policy. The voting policy was described as follows: a protein was classified as middle-confidence if it is included in MitoP2 or MitoCarta dataset, or was identified from more than five proteomic experiments, while the remaining were low-confidence. The high-confidence mitochondrial proteins were strongly supported by the 23 datasets, which may represent the most common proteins in mitochondria. Some other proteins however may intermittently bind to the surface of mitochondria, making it hard to discover by mass spectrometry, thus may fall into the middle-confidence or even low-confidence category. Nevertheless, by integrating sufficient datasets from various experimental conditions, the risk for the latter case will drop a lot. Considering the fact that some proteins may expressed under certain circumstances or special tissues, the information for tissue/organ origin of a protein was retained for researchers’ judgments on our web pages. The 1124 high-confidence proteins as well as the 1159 middle-confidence proteins together made up the MitoCom dataset.

**Figure 2:**
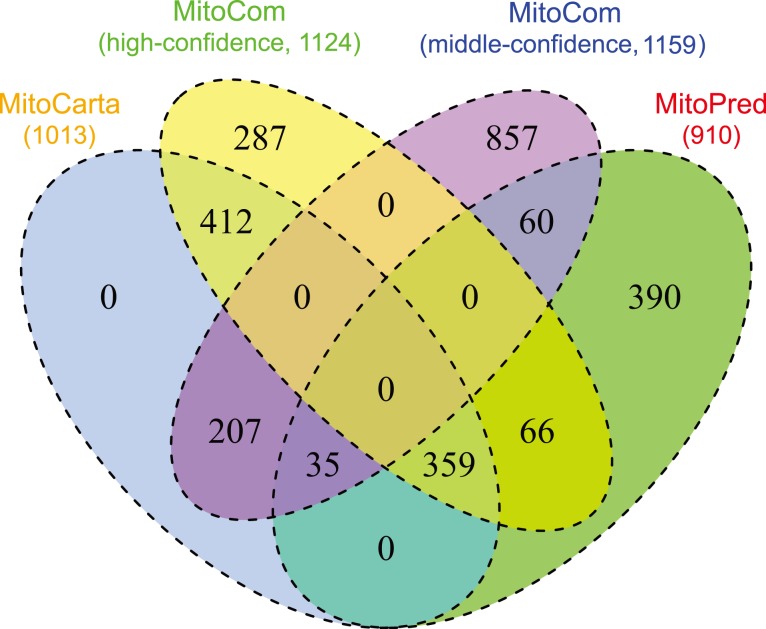
Venn diagram of the four datasets: MitoCom (high-confidence), MitoCom (middle-confidence), MitoCarta and MitoPred.

To evaluate the quality of MitoCom, a comparison between MitoCom (high-confidence proteins) and two mitochondrial databases, MitoPred [53] and MitoCarta, was carried out by using the MitoP2 dataset as the reference set. As shown in table 2, the high-confidence proteins in MitoCom showed considerable overlap with MitoPred and MitoCarta, meanwhile it retained a wider coverage, greater sensitivity and lower false discovery rate, which can reduce the “noise” in high-throughput mammalian mitochondrial protein identification effectively. The venn diagram (figure 2) between these three datasets and the middle-confidence proteins showed that the high-confidence proteins had about 74% overlap with MitoCarta and MitoPred, while keeping 288 proteins that identified uniquely by this work. The high-confidence proteins in MitoCom extended the mitochondrial proteome while the middle-confidence proteins can be a clue for a more complete mitochondrial proteome. Thus, our inventory of mammalian mitochondrial proteins would be more comprehensive and accurate in comparison to other databases, which enables it to be a powerful tool for mitochondrial proteome studies.

**Table 2:** Quality comparison of MitoCom with other mitochondrial databases.

Database	Number	Sensitivity	False discovery rate
MitoCom*	1109	97.34%	11.30%
MitoCarta	1013	86.10%	13.70%
MitoPred	910	50.10%	14.80%

*Just the high-confidence proteins.

### Functional linkages among mitochondrial proteins

With the rapidly increasing number of discovered mitochondrial proteins, a critical task beyond protein identification is to annotate cellular functions for newly-identified mitochondrial proteins and to associate their functional roles with human mitochondrial disorders. We have pursued these goals by integrating genomic features from heterogeneous data sources to build quantitative functional links among mitochondrial proteins. Since a single data source usually reflects only one type of functional association between proteins (genes), and its coverage is relatively limited, functional associations from multiple data sources should be jointed to achieve larger coverage and better accuracy.

In the previous step, we have built an inventory of 1124 mammalian mitochondrial proteins. This yielded 631688 potential mitochondrial protein-protein functional linkages. To validate these protein pairs, we systematically combined 11 genomic features about 15 datasets (Table 3) using machine learning algorithm.

**Table 3:** Functional features for mammalian mitochondrial FLN construction.

Functional features	Data sets	Description	Scale	Data source
Protein-protein interaction		Protein-proteininteraction.	Genome-scale	HPRD [54], I2d [55]
Domain-domain interaction		Protein pairs haveinteracting proteindomains.	Genome-scale	3did [56]
Shared domains		Proteins pairs sharingsame protein domains.	Genome-scale	Interpro [57]
Genomic context	Rosetta Stone	Gene fusion events.	Genome-scale	Prolinks [58]
	Phylogenetic profiles	Phylogenetic Profiles [59]of 1086 genes among 600species.(Table S6)	Genome-scale	NCBI, KEGG [60]
Genetic interaction		Mutations in two genesproduce a phenotype thatis greatly different fromeach mutation’sindividual effects.	Genome-scale	Saccharomyces Genome Database [61]
Phenotypic semantic similarity		Sementic simlilarity ofmouse phenotypicterms.	Genome-scale	Mammalian Phenotype Browser [62]
Co-expression	GSE1133 [63]	Gene expression profile ofthe vast majority ofprotein-encoding humanand mouse genes in79human and 61 mousetissues.	Genome-scale	GEO [64]
	GSE4726 [65]	A quantitative andcomprehensive atlas ofgene expression in mousedevelopment.	Genome-scale	GEO
	GSE4330 [29]	Microarray time-course ofmouse myotubestransducedwith thetranscriptionalco-activatorPGC-1α, whichis known toinducemitochondrial biogenesisin muscle cells.	Mitochondria-specific	GEO
	GSE6210 [66]	Gene expressionprofile in livertissue andquadricepsmuscle in mice betweencontrol and the PCG-1βmutant, a transcriptionalcoactivator thatpotently stimulatesmitochondrialbiogenesis andrespiration of cells.	Mitochondria-specific	GEO
GO semantic similarity		GO Sementicsimilarityof genessharing thesame biologicalprocess terms	Genome-scale	The GeneOntology [16]
Protein expression profiles		Mitochondrialproteinprofiles of protein-coding genes inheart,brain, liver, kidneyand lung.	Mitochondria-specific	Results of ThomasKislinger et al [36]
Disease involvement		A pair ofgenes thatannotated in thesamedisease.	Mitochondria-specific	OMIM [67]
Operon		Operon data of*Rickettsia* *prowazekii.*	Mitochondria-specific	Database of prOkaryotic OpeRons [68]

The integrated features were shown as follows:

- **Protein-protein interaction (PPI).** Protein-protein interactions are fundamental to all biological processes. The interacting proteins should have closely functional association.
- **Domain-domain interactions.** Proteins perform their biological functions often through domains as units. Thus two proteins may have similar function if they contain domains with capability of interacting.
- **Shared domains.** As well known, domain is the functional unit in protein. Hence, proteins possess the same set of domains should have similar function.
- **Genomic context.** Genomic context including phylogenetic profiles and Rosetta Stone can be powerful evidence for functional linkages between genes. Gene pair that has similar phylogenetic profile or appears in a gene fusion event tends to be functionally associated [69], [70].
- **GO Semantic Similarity.** Gene ontology defines a gene function with a hierarchical structure in three dimensions including cellular component, molecular function and biological process. Two genes with terms that share the same parent far from root should be functional associated [24]. Thus, the GO semantic similarity can be used to measure function association between genes.
- **Genetic interaction.** Genetic interactions, such as synthetic lethal and synthetic growth, infer those involved genes have strong correlation. These correlations are also evidences of functional associations.
- **Phenotypic semantic similarity.** Genes leading to similar phenotypes should have functional linkages, as similar phenotypes may need similar substances or involve similar processes.
- **Gene co-expression.** Genes encoding proteins that are involved in the same process are expected to be simultaneously expressed in time and space [71]. Therefore, genes with similar expression patterns should have related function. To profile gene expression, four microarray datasets were selected. GSE1133 and GSE4726 interrogate the expression of the vast majority of protein-encoding human and mouse gene that can give us a global view on gene expression profile at the genome scale, while GSE4330 and GSE6210 studied the influence of mutant in PGC1α and PGC1β, both of which are transcriptional coactivator that potently stimulates mitochondrial biogenesis and respiration of cells, focusing on mitochondrial-specific genes.
- **Proteomic profiles.** Similar to gene co-expression profile, proteomic profile may lead to better understanding of mitochondrial feature at protein level. Thomas Kislinger et al [36] examined the protein content of four organellar compartments in six mouse organs, which could be a valuable resource. We extracted the mitochondrial-specific proteomic profile from this dataset.
- **Diseases involvement.** Genes annotated in the same disease tend to have functional associations.
- **Operon.** Based on the endosymbiotic theory, mitochondrion may evolve from an ancestor of *Rickettsia prowazekii*, which shares a lot of homological genes with mitochondrial genome [72]. As a functional unit, operon contains a series of genes that involved in same biological process. Therefore, mitochondrial genes whose homologies appear in the same operon in *Rickettsia prowazekii* should be an evidence for functional associations.

To implement the machine learning algorithm, a GSP and a GSN were first constructed (see materials and methods). Based on the well-defined GSP and GSN, we investigated the coverage of each genomic feature, revealing that several datasets had very low coverage (<20%). Only five datasets including GO semantic similarity, gene co-expression, proteomics profiles and phenotypic semantic similarity covered over 20% on the GSP and GSN (Table S3). For integrating these datasets, we used a naïve Bayes classifier [73], [74] owing to its two advantages. First, it can integrate heterogeneous kinds of evidence and tolerate missing data among them. Second, it is simple but highly efficient to tackle data in a large scale with short time consumption.

As a prerequisite for using naïve Bayes classifier, all the datasets should be conditionally independent. We assessed the statistical independence between each pair of datasets with coverage more than 20% by calculating the PCC. As shown in Table S4, these datasets are relatively independent with the maximum PCC is only 0.217. Following the naïve Bayes theorem, a likelihood ratio (LR) corresponding to a specific biological evidence could be used to measure the predictive power or confidence degree. Thus we measured the power of individual datasets to infer functional linkages by using the naïve Bayes model. Each dataset was divided into several bins, and then the LR for each bin was calculated according to the GSP and the GSN. As shown in Figure S1, all the 15 datasets were clearly correlated with LRs and all the datasets had one or more bins with LR>1, which suggested that the 15 datasets can be used to infer functional linkages between genes.

**Figure 3:**
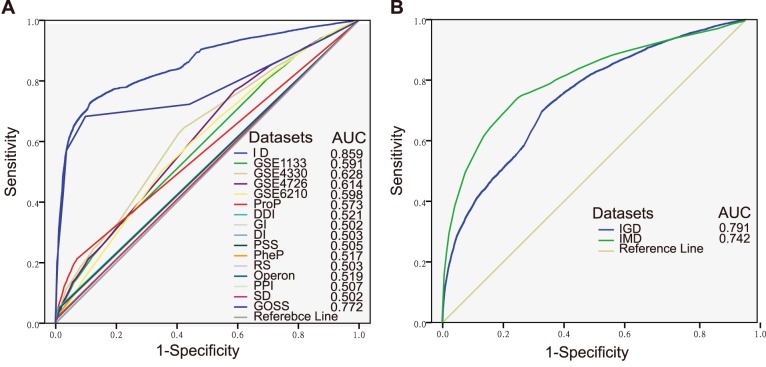
ROC curves for evaluating the performances of various data sources using cross-validations. (A) ROC curves and AUC of individual dataset and integrated dataset. The data sources are highlighted in different colors. (B) ROC curves and AUC of mitochondrial-specific (green) and genome-scale (blue) datasets. ID: Integrated datasets; ProP: Protein expression profiles; DDI: Domain-Domian Interaction; GI: Genetic Interaction; DI: Disease Involvement; PSS: Phenotypic Semantic Similarity; PheP: Phylogenetic Profiles; RS: Rosetta Stone; PPI: Protein-Protein Interaction; SD: Shared Domains; GOSS: GO Semantic Similarity; IGD: Integrated Genomic-scale Datasets; IMG: Integrated Mitochondrial-specific Datasets; ROC: receiver operating characteristic; AUC: area under ROC curves.

To evaluate the performances of individual dataset model and integrated model, we carried out five-fold cross-validation and drew the receiver operating characteristic (ROC) curve (Figure 3A). The figure showed that the integrated model had the largest area under ROC curves (AUC), demonstrating the superiority of data integration. The results also suggested that individual data models have limited capability to correctly identify functional linkages between genes. Most of individual dataset models including gene co-expression model and proteomic profile model have similar performances with an AUC around 0.6, much lower than the integrated model. The rest datasets except for the GO semantic similarity model showed no difference to the reference line, indicating their inefficiency. A clear exception was GO semantic similarity model, which had an AUC of 0.772, a little lower than the integrated data. The GSP and GSN were derived from prior knowledge, which will introduce in bias when estimating the GO semantic similarity model that was also derived from prior knowledge. If we use this model to predict novel function linkage, the prediction ability is limited. Therefore, we can conclude that data integrating approach is the best when try to predict novel functional linkages.

Furthermore, we classified the 15 datasets as genomic-scale and mitochondria-specific according to dataset source and data scale. A dataset was considered as mitochondria-specific if the dataset was generated from an experiment was aimed at mitochondrial study, like GSE4330, GSE6210 and proteomic profile, If a dataset contains information only derived from the mitochondrial proteome, such as diseases involvement, operon and GO semantic similarity, it was also considered as mitochondria-specific. As shown in Figure 3B, the integrated mitochondria-specific model had a larger AUC than the integrated genome-scale model, which indicated that the mitochondria-specific dataset was more powerful to construct FLN.

**Figure 4:**
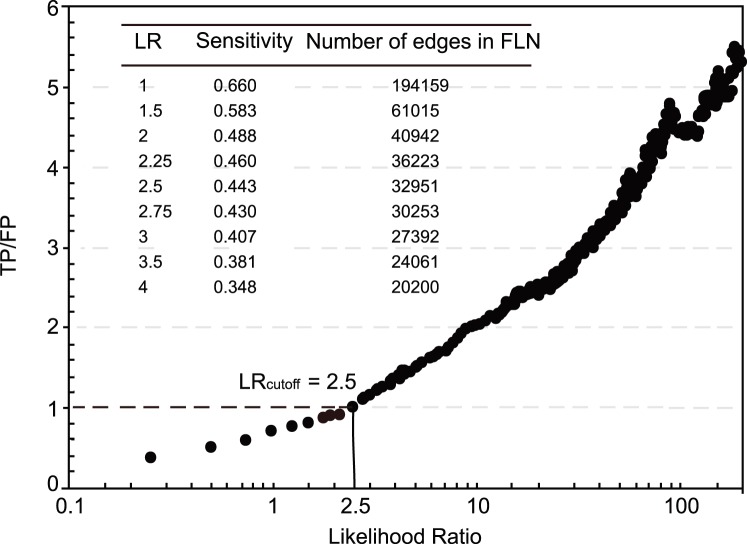
TP/FP ratios vs. LR cutoff, and corresponding sensitivity. TP: True Positive; FP: False Positive. Sensitivity = TP/(TP+FN).

After data integration, each protein pair has been attached a LR score. A cutoff of LR was determined afterward, which representing as an indicator of whether a protein pair is functional associated (that is, yes if the composite LR is above the LR cutoff, no if not). We used the ratio of true positive (TP) to false positive (FP) to measure the prediction accuracy, and plotted the TP/FP ratio as a function of LR cutoff (Figure 4). We found that there is an apparent positive correlation between the TP/FP ratio and LR cutoff, but the sensitivity decreases monotonically and the FLN scale shrinks simultaneously with the increase of LR cutoff. A composite LR cutoff of 2.5 was selected where the TP/FP ratio was 1, which means that we can achieve 50% prediction accuracy at this resolution. Based on this LR cutoff, the resulting FLN is comprised of 1072 proteins (covering approximately 71% of the mitochondrial proteome) and 32951 weighted functional linkages (Table 4), the average number of functional linked neighbors per protein is 61. The mitochondria-specific FLN owns such high coverage and linkage density, which is essential to the successful utilization of the FLN for disease gene prediction and prioritization.

**Table 4:** Descriptions and parameters of four networks.

	Description	NumberofNodes	NumberofEdges	Averagenumber ofneighbors	Density
FLN	FLN among the proteinswith high confidence	1072	32951	61.476	0.057
FLNhm	FLN among the proteinswith high or middle confidence	1992	1983036	1991.000	1
PPI network	Protein-protein interactionsnetwork derived from HPRDand I2D	1322	9049	12.850	0.01
Co-expressionnetwork	Co-expression networkderived from microarrayexperiment GSE1133	1684	1417186	1683.000	1

### Disease candidate gene prioritization

With the FLN, we aimed at using the information to prioritize candidates for mitochondrial diseases. The utility of FLN for disease candidates prioritization based on the assumption that genes underlying the same or related diseases tend to be functionally related [69]. Based on this assumption, FLNs have been successfully used to identify novel disease genes in recent studies [74]–[76]. Meanwhile, many network-based methods have been developed to prioritize candidates, for example, random walk, neighborhood-based and diffusion kernel methods. These methods mostly locate the known disease genes in network as “seeds” first, and then score the associated neighborhoods of these seeds by specific algorithm, and finally candidates are prioritized based on the scores of candidates.

In this work, four network-based methods were chosen for disease candidate prioritization. The average adjacency ranking (AAR) rule has been successfully used by Guan Y et al. to predict novel pathway components [74]. PageRank with Priors (PRP), K-step Markov (KSM) and Heat Kernel Diffusion Ranking (HKDR) methods were also used to prioritize disease candidates based on PPI networks [75]. Goncalves et al analyzed the performance of the four methods, indicating their applicability in prioritizing disease candidates [76].

Despite the impacts of ranking approaches, FLN should outperform the single source networks for the reason that multiple evidence increases coverage/density and reduces bias toward individual sources [76]. We evaluated the effectiveness of the four ranking algorithms utilizing the FLN and two single source networks including PPI network and co-expression network to prioritize candidates, both of which were derived from single data source. Furthermore, because the ranking algorithms are also susceptible to the network scale and density, the FLN was expanded into a scale-larger network named the FLNhm by including the middle-confidence mitochondrial genes and their functional linkages (the LR cutoff wasn’t used). We downloaded the disease data from the OMIM database, and extracted those that have at least two OMIM-annotated disease genes present in the networks for identifying disease candidates. Owing to the scale difference, different sets of mitochondrial diseases and disease genes were analyzed when utilizing the four networks respectively. Using known disease-associated genes as “seeds”, Leave-one-out cross-validation tests were conducted. ROC curves were plotted to visualize the performance with AUC values as quantitative measures.

**Figure 5:**
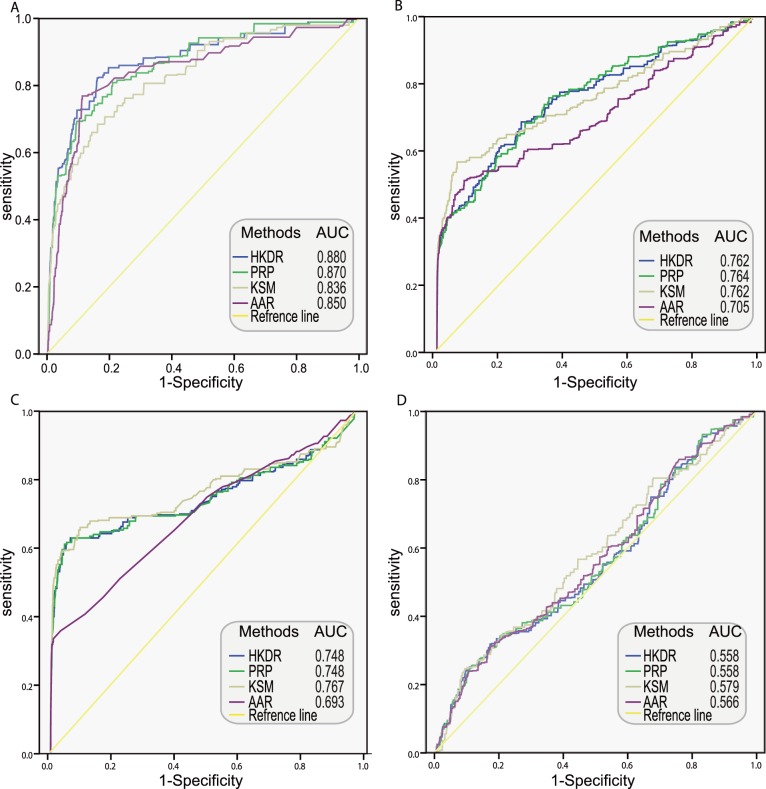
ROC curves for evaluating the performances of four networks on disease-gene prioritization. (A) The ROC curve for FLN. (B) The ROC curve for FLNhm. (C) The ROC curve for PPI network. (D) The ROC curve for co-expression network. AAR: Average Adjacency Ranking; PRP: PageRank with Priors; KSM: K-Step Markov; HKDR: Heat Kernel Diffusion Ranking; FLN: Functional Linkage Network among high-confidence mitochondrial proteins; FLNhm: Functional Linkage Network among high-confidence and middle-confidence mitochondrial proteins; PPIN: Protein-Protein Interaction Network; CEN: Co-Expression Network.

For the reason that algorithms performance differently with the parameter set and the scale of network different, different test parameter sets were empirically selected to decide the best algorithm and its optimal parameter set for each network. (see materials and methods).We decided the optimal parameters of the algorithms on each network based on the AUC (Table S5). Figure 5 showed ROC curves of the four algorithms with optimal parameters on the four networks. The HKDR, PRP and KSM algorithms outperformed neighborhood algorithm AAR, which indicated that the three algorithms utilizing the whole topology information were superior to algorithms utilizing local topology information. It may be the result of that the algorithms that utilize the whole topology can compensate for missing links by exploiting higher order neighborhoods and path redundancies [76]. HKDR and PRP algorithms performed best respectively on the FLN and the FLNhm. KSM had a poor performance compared with PRP and HKDR on FLN and FLNhm, but outperformed the two algorithms on the PPI network and the co-expression network, suggest that KSM algorithm was better in compensating for missing links than HKDR and PRP algorithm when being utilized in single source networks.

Furthermore, we also observed that the performances of the four algorithms dropped orderly and significantly in FLN, FLNhm, PPI network and co-expression network. As a single source network, the PPI network and co-expression network were supported to be less informative with limited coverage and large number of false positive linkages. Therefore, PPI network and co-expression network performed worse than FLN and FLNhm as expected. The FLNhm, which was denser and with bigger coverage than FLN, but performed worse than FLN, indicated that topology also play an important role in the performance of network. Being the best performance of cross-validation, HKDR algorithm with its optimal parameter (n = 3) on the FLN were chosen to rank candidates of mitochondrial diseases.

### Mitochondrial complex I deficiency: a case study

Mitochondrial complex I deficiency, the most common cause of mitochondrial disorders (accounts for ∼30% cases of respiratory chain deficiency in humans) [77], causes a wide range of clinical disorders, ranging from lethal neonatal disease to adult-onset neurodegenerative disorders. Phenotypes include macrocephaly with progressive leukodystrophy, nonspecific encephalopathy, hypertrophic cardiomyopathy, myopathy, liver disease, Leigh syndrome, Leber hereditary optic neuropathy, and some forms of Parkinson disease. It shows extreme genetic heterogeneity. Up to now, mutations in 17 genes encoding mitochondrial complex I subunits have been described in the OMIM database. However, these 17 genes account for disease in only a minority of mitochondrial complex I patients. Since mitochondrial complex I has at least 45 subunits [78], [79], mutations in any of the other approximately 30 supernumerary subunit genes could potentially cause mitochondrial complex I deficiency, even mutations in other genes functionally associated with mitochondrial complex I subunits are also possible causes. Here, heat diffusion was applied to rank and screen promising candidates of mitochondrial complex I deficiency based on linkage with known disease genes, then we assessed the ability of prioritization to identify unknown causes.

**Figure 6:**
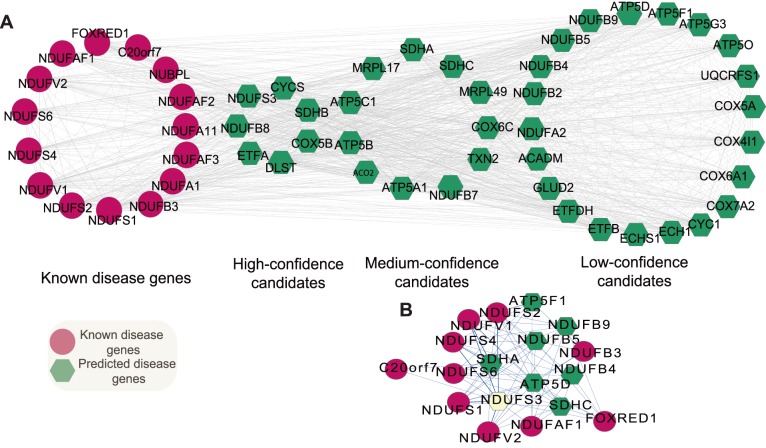
Prioritization results for mitochondrial complex I deficiency. (A) A hypothetical FLN of mitochondrial complex I deficiency. The FLN is comprising of known disease genes (highlighted in red) annotated in OMIM and predicted disease genes (highlighted in greed). The candidates are classified into three levels (high-confidence, middle-confidence and low-confidence) according to their ranking scores. (B) The functional linkage sub-network among the candidate NDUFS3 that has a top score on ranking algorithm for mitochondrial complex I deficiency.

Fifteen of these disease causing genes are present in our function linkage network. The importance of each gene in the function linkage network relative to mitochondrial complex I deficiency was ranked using these 15 genes as seeds. We investigated the top 15 candidates (Table 5), almost all of which could be associated with mechanisms of mitochondrial complex I deficiency (Figure 6). In the top three, the *NADH dehydrogenase 1 beta subcomplex, 8, 19 kDa* (*NDUFB8*) is known to encode a subunit of mitochondrial complex I [79], [80]. Haack *et al*. found mutations in *NDUFB8* result in decreased activity and amount of mitochondrial complex I [81]. And the *cytochrome c oxidase subunit Vb* (*COX5B*), known to cooperate with mitochondrial complex I in respiratory electron transport chain, is a terminal enzyme of the mitochondrial respiratory chain [82]. *Electron-transfer-flavoprotein, alpha polypeptide* (*ETFA*), in the third place, shuttles electrons between primary flavoprotein dehydrogenases and the membrane-bound electron transfer flavoprotein ubiquinone oxidoreductase [83]. Mutations in *ETFA* are causative for multiple acyl-CoA dehydrogenase deficiency, and result in decreased activity of mitochondrial complexes I [84], [85]. It is worth noting that the *NADH dehydrogenase Fe-S protein 3, 30 kDa* (*NDUFS3*), ranked 4th, encodes one of the iron-sulfur protein components of mitochondrial NADH: ubiquinone oxidoreductase (complex I) [79], [80]. Bénit *et al*. found mutations in *NDUFS3* related to isolated mitochondrial complex I deficiency by using a combination of denaturing high performance liquid chromatography and sequence analysis [86]. Haack *et al*. also reported pathogenic mutations in *NDUFS3* caused isolated mitochondrial complex I deficiency by combining unbiased exome analysis, sequential filter, and functional investigation [81]. The *NADH dehydrogenase 1 beta subcomplex, 7, 18 kDa* (*NDUFB7*), ranked 14th, encodes a subunit of mitochondrial complex I [79], Triepels *et al*. found pathogenic mutations in *NDUFB7* in the patients of mitochondrial complex I deficiency [87].

**Table 5:** The 30 top-ranking genes for mitochondrial complex I deficiency.

Ranking	Score	GeneID	Symbol	Description
1	0.802272	4723	NDUFV1	NADH dehydrogenase flavoprotein 1, 51 kDa
2	0.697647	51103	NDUFAF1	NADH dehydrogenase 1 alpha subcomplex, assembly factor 1
3	0.691345	4694	NDUFA1	NADH dehydrogenase 1 alpha subcomplex, 1, 7.5 kDa
4	0.688717	4726	NDUFS6	NADH dehydrogenase Fe-S protein 6, 13 kDa
5	0.686216	4719	NDUFS1	NADH dehydrogenase Fe-S protein 1, 75 kDa
6	0.685317	4720	NDUFS2	NADH dehydrogenase Fe-S protein 2, 49 kDa
7	0.68423	4709	NDUFB3	NADH dehydrogenase 1 beta subcomplex, 3, 12 kDa
8	0.681527	4729	NDUFV2	NADH dehydrogenase flavoprotein 2, 24 kDa
9	0.676788	4724	NDUFS4	NADH dehydrogenase Fe-S protein 4, 18 kDa
10	0.65894	79133	C20orf7	chromosome 20 open reading frame 7
11	0.656693	126328	NDUFA11	NADH dehydrogenase 1 alpha subcomplex, 11, 14.7 kDa
12	0.656337	91942	NDUFAF2	NADH dehydrogenase 1 alpha subcomplex, assembly factor 2
13	0.656292	55572	FOXRED1	FAD-dependent oxidoreductase domain containing 1
14	0.656166	25915	NDUFAF3	NADH dehydrogenase 1 alpha subcomplex, assembly factor 3
15	0.656105	80224	NUBPL	nucleotide binding protein-like
16	0.115148	4714	NDUFB8	NADH dehydrogenase 1 beta subcomplex, 8, 19 kDa
17	0.109928	1329	COX5B	cytochrome c oxidase subunit Vb
18	0.090152	2108	ETFA	electron-transfer-flavoprotein, alpha polypeptide
19	0.087915	4722	NDUFS3	NADH dehydrogenase Fe-S protein 3, 30 kDa
20	0.083753	6390	SDHB	succinate dehydrogenase complex, subunit B, iron sulfur (Ip)
21	0.078834	1743	DLST	dihydrolipoamide S-succinyltransferase (E2 component of 2-oxo-glutarate complex)
22	0.070645	54205	CYCS	cytochrome c, somatic
23	0.068273	509	ATP5C1	ATP synthase, H+ transporting, mitochondrial F1 complex, gamma polypeptide 1
24	0.067436	506	ATP5B	ATP synthase, H+ transporting, mitochondrial F1 complex, beta polypeptide
25	0.06552	1345	COX6C	cytochrome c oxidase subunit VIc
26	0.061017	25828	TXN2	thioredoxin 2
27	0.060686	6391	SDHC	succinate dehydrogenase complex, subunit C, integral membrane protein, 15 kDa
28	0.060526	50	ACO2	aconitase 2, mitochondrial
29	0.060351	4713	NDUFB7	NADH dehydrogenase 1 beta subcomplex, 7, 18 kDa
30	0.058394	740	MRPL49	mitochondrial ribosomal protein L49

Despite continued progress in our understanding of the molecular basis of mitochondrial complex I deficiency, the genetic defect remains elusive in many cases. With the application of the function linkage network, potential pathogenic causes could be ranked and prioritized. Furthermore, top ranked candidates could guide the design of new disease-genes association studies and offer clues for new treatment strategies.

### Database and web server

We constructed a database named MitoProNet for storing our results including mammalian mitochondrial proteins, the FLN and human disease information. MitoProNet is an object-relational database implemented by mysql accessible via a user-friendly web interface written in JSP.

**Figure 7:**
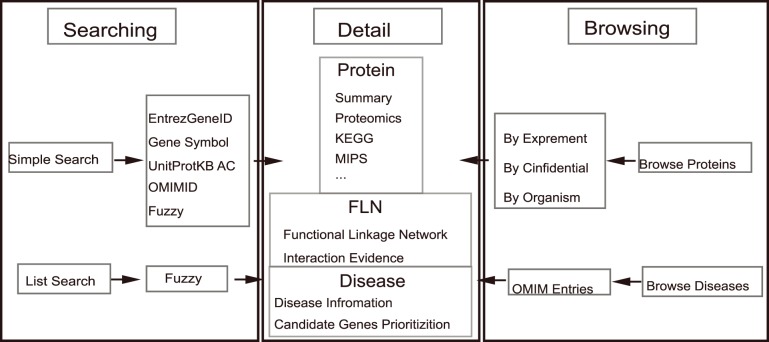
System architecture and main contents of MitProNet. MitProNet is composed of three sections including mitochondrial protein part lists, annotations of mitochondrial protein and disease information.

**Figure 8:**
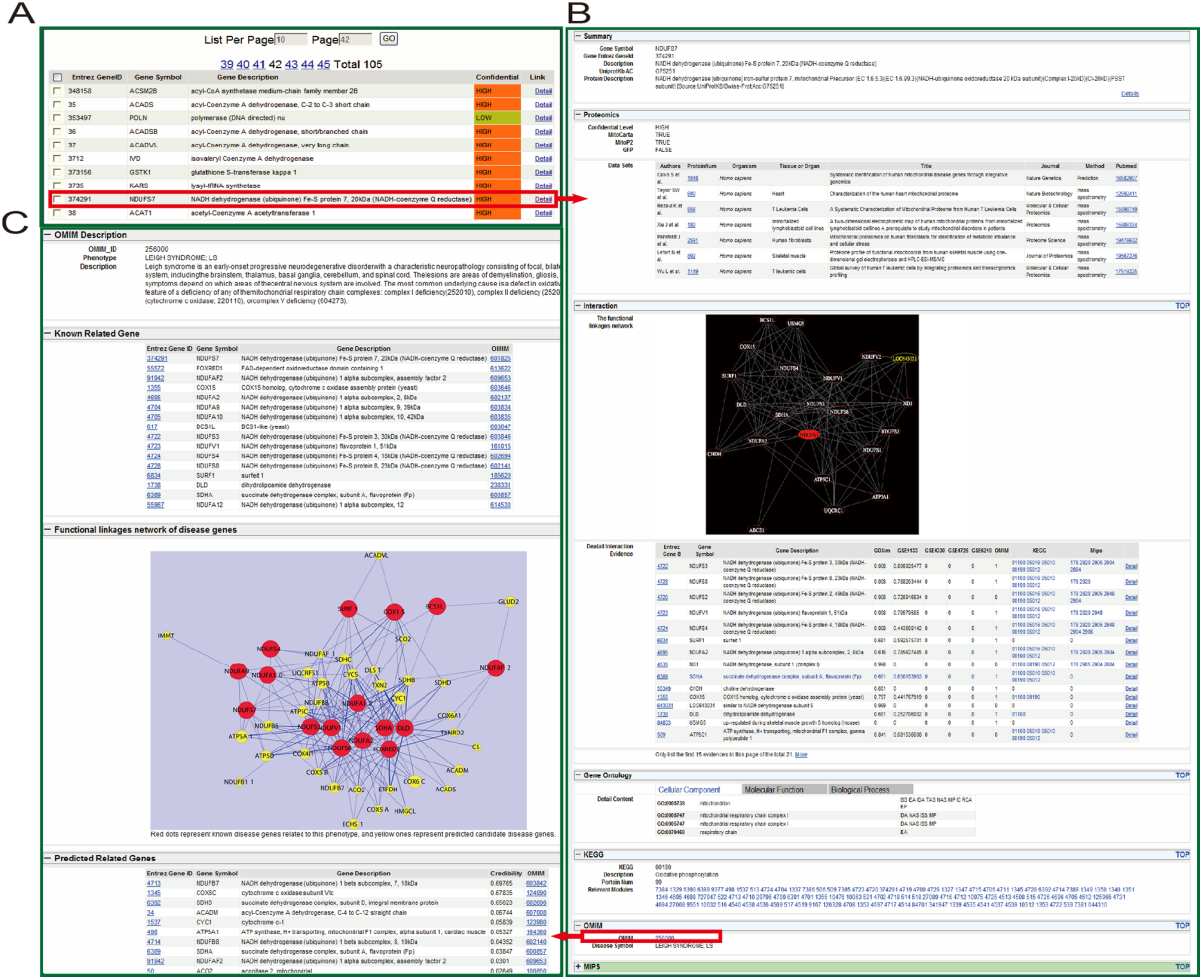
Web pages in MitProNet. (A) A list page of mitochondrial proteins. The mitochondrial proteins can be listed according to proteomic datasets, confidence levels and organisms, respectively. (B) The outcome page for the query protein NDUFS7, an annotated disease gene for Leigh syndrome. The page provides a brief summary of the query protein, subcellular localization evidences and a FLN among the query protein. Moreover, the query protein is annotated according to the information collected from their original sources including GO, KEGG, MIPS and OMIM. (C) The prioritization results for Leigh syndrome. The result page includes a brief description for this phenotype, disease genes and a FLN among these genes. The disease genes are listed dividedly as the known genes and the candidates that are ordered by these ranking scores.

The main contents of MitProNet are demonstrated in Figure 7 including proteome section, disease section and FLN among proteins or genes, which could be accessed by browsing or searching in MitProNet. Users can browse proteome data and disease data by clicking the proteome interface and the disease interface. The proteome interface provides comprehensive data of mammalian mitochondrial proteins that were identified experimentally. Results could be displayed orderly according to experiment, confidence level or organisms. The disease interface provides comprehensive information about typical mitochondrial diseases, including description, known disease genes, top ranking disease candidates ranked in our study, as well as functional linkages network among these genes. Users can also click the name of a protein of interest, the results include description of the protein and its annotation information will be displayed via HTML pages. Moreover, a local functional linkages network can be visualized online as a scalable vector graphics (SVG) file, which provides the means for a fast visual evaluation of the protein’s functional association with other proteins. The search interface also allows users to source the proteins or diseases of interest conveniently by using a variety of keywords include gene IDs, gene symbols, protein IDs and OMIM IDs. And Figure 8 showed a case of browsing and searching in MitProNet. All these data presented in MitProNet can be downloaded freely through our download interface.

## Conclusions

In our work, we carried out a comprehensive mammalian mitochondrial proteomic study through a three-step approach. We compiled an extensive inventory of mammalian mitochondrial proteins by combining 23 genomic-scale datasets. Our inventory showed considerable overlap with MitoPred and MotoCarta, the two best existing mitochondrial databases, but held greater sensitivity and lower false discovery rate. The high-confidence proteins along with the middle-confidence proteins provide a narrowed scope of candidates for mitochondrial proteins with relatively high possibility. We also constructed a comprehensive and high quality weighted FLN among mitochondrial proteins through integrating 15 heterogeneous functional features. With the comprehensive features integrated, the FLN is less biased towards single evidence and can be more accurate and with higher coverage. The high coverage and linkage density is essential to the successful utilization of the FLN for disease gene prediction and prioritization. Thus the FLN we presented can provide valuable resource for researches on mammalian mitochondrial proteomics. One important utility of the FLN is for mitochondrial disease genes predicting and prioritizing. The top-ranking candidates for the mitochondrial diseases reported in this work represent the highly possible risk genes for the specific disease, which provide a narrowed spectrum of suspects for these important human diseases and will promote the disease-genes association studies and offer clues for new treatment strategies. Moreover, with the identification of new disease genes, these results can be further integrated into our framework for better disease gene predictions. Furthermore, a web-based database MitProNet was also implemented. Researchers can easily locate a gene of interest and analyze those tightly associated genes. The visualization of local FLN around the gene can be a rapid and convenient approach to inspect the relationship of those associated genes. The disease related network present an overall landscape of the relationship of known and candidate genes. The complete set of mitochondrial genes and FLN are also provided. Thus the FLN and the disease candidates implemented in MitoProNet would facilitate the researches in mitochondria and diseases related to this important organelle.

## Materials and Methods

### An inventory of mammalian mitochondrial proteins

To reduce redundancy, the proteins were transformed into corresponding genes identified unique by Entrez GeneID.

#### Gold standard sets

The GSP dataset was comprised of human mitochondrial proteins that were curated from the MitoP2 database [88]. To avoid contamination, we only used proteins with supports of sublocalization experiments, and excluded those characterized solely by large-scale proteomic studies. The GSN, on the other hand, was selected from proteins located in other cellular compartments according to Gene Ontology (GO) annotations. For those proteins with multiple subcellular locations, we excluded those with subcellular location in mitochondrial components or locations from the GSN. As a result, the GSP dataset contained 553 proteins, while the GSN dataset consisted of 9950 non-mitochondrial proteins.

#### Cross validation and evaluation of machine-learning algorithms

When training the classifiers, the 23 proteomic experiments datasets were considered as ‘features’. And for each feature, we assigned a score 1 to each human gene product if the product exists in the dataset, or 0 otherwise. We used the 10-fold cross validation to evaluate prediction performance of these machine-learning classifiers [89]. For each machine-learning classifier, at first, both the GSP and GSN were randomly partitioned into ten equal-sized folds. After that, the machine-learning classifier was trained on nine folds and the remaining one fold was used as a test set to identify the number of positives and negatives. This was repeated ten times with a different fold used for testing each time.

#### Calculating sensitivity and false discovery rate

Sensitivity is defined as TP/(TP+FN), where TP is the number of true positives and FN is the number of false negatives, respectively, estimated from gold-standard sets. The false discovery rate (FDR) is the proportion of all predictions that are false; FDR = FP/(FP+TP), where FP represent the number of false positives [29].

### Construction of mitochondrial FLN through data integration

To carry out the construction of FLN, each dataset should be transformed into protein pairs with functional linkage. The preprocessing is described in supplementary methods (Method S1) in detail.

#### Gold standard sets

In this study, we downloaded KEGG pathway [60] and MIPS complex [90] about mitochondrion. The GSP were defined as mitochondrial protein pairs sharing the same KEGG pathway or existing in the same MIPS complex, while the GSN were defined as mitochondrial protein pairs both annotated by KEGG pathway or MIPS complex terms but that do not share any term.

#### Naïve Bayes for mammalian mitochondrial FLN construction

According the Bayesian theorem, the prior odds (*O_prior_*) of finding a gene pair with functional linkage could be calculated as:
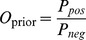
(1)where *P_pos_* is the probability that a gene pair functionally relates within all the possible gene pairs while the *P_neg_* stands for the probability that a gene pair isn’t functionally related. When considering the given *n* evidences (*E*) that stands for the functional features, the posterior odds (*O_posterior_*) of a functional linkage gene pair could be computed as:
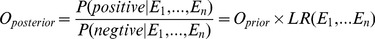
(2)where *LR(E_1_,…,E_n_)* is the likelihood ratio of the *n* evidences(*E*). From Equation 1 and Equation 2, the LR could be calculated as:



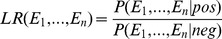
(3)If we assume that the evidences are conditionally independent, the composite *LR* can be calculated simply as following:
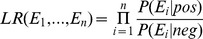
(4)


And Equation 4 can also be written as the following:
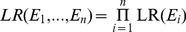
(5)


#### Cross validation and cutoff selection

We employed the five-fold cross-validation against the golden standard datasets to evaluate the overall prediction performance under different LR cutoffs. First, both the GSP and GSN datasets were randomly partitioned into five equal-sized folds. After that, the naïve Bayesian classifier was trained on four folds and the remaining one fold was used as a test set to identify the number of positives and negatives. This was repeated five times with a different fold used for testing each time. We used the ratio of true positive to false positive (TP/FP) and the sensitivity to measure the prediction accuracy.

### Ranking the mitochondrial disease gene

#### Average Adjacency Ranking

Given a particular mitochondrial disease, firstly, *m* genes were extracted randomly from known disease-related mitochondrial genes as seed gene set, and the rest of the genes were treated as unknown ones. Then for every other gene, we compute the adjacency to the *m* seeds. This process was repeated one hundred times with random samplings of the seed set. Lastly, we calculated the average adjacency with a given disease for each gene:
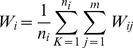
(6)where *W_i_* represents the weight of each gene associate with a given disease and *j* represents the seed genes, and *W_ij_* is the functional linkage weights connecting gene *i* and seed gene *j*. *n_i_* is the number of times gene *i* was not a member in the seed set and *k* is the iteration number.

#### PageRank with Priors

PRP mimics a random jump procedure in network, which start with known disease-related genes and randomly jump to candidate genes. When the system jump to a candidate gene, system can continue jumping to other candidate genes or jump back to known disease-related genes and then restart the procedure. After enough jumping, PRP scores each candidate gene based on the probability that system jump to the gene. The iterative stationary probability is:
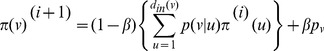
(7)where *p_v_* represents the “prior bias” which means the probability to start with a particular genes. *p_v_* = 1/|R| if v in root node set R (known disease-related gene set); *p_v_* = 0 otherwise. *β* is empirically defined on [0, 1], represents a “back probability” which means the probability to jump back to the root node in each step. d_in_ *(v)* is the in-degree of *v*. *p(v|u)* is the probability of arriving node *v* from *u*.

#### K-step Markov

KSM also mimics a random jump procedure that start with disease-related genes and ends after fixed K steps. It computes the relative probability that the system will spend time at any particularnode given that it starts in a set of roots R and ends after K Steps [91]. K keeps a balance between distributions of candidate genes ‘biased’ toward known disease-related genes. With a larger *K*, system gets a more steady distribution of candidate genes [75]. The to compute the K-Step Markov importance is:
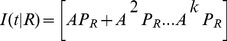
(8)Where *A* is the transition probability matrix of network, *p_R_* is an vector of initial probabilities for the root set *R* (known disease genes set), *k* is the probability transition steps and *I(t|R)* is the *t-th* entry in this sum vector.

#### Heat Kernel Diffusion Ranking

The Heat Kernel Diffusion Ranking approach ranks the candidate genes by diffusing the signal of ‘seeds’ to the candidate genes through the network based on the weighted edges [92]. The network can be represented as a weighted, simple graph G, where genes are nodes and weighted linkages are weighted edges. Given a graph *G*, let A be the Adjacency matrix where *a_ij_ = w_ij_* and then *D* can be defined as 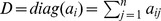. The transition probability matrix *W* of a random walk on *G* is defined as *W = D^−1^ A*. Conside*r L = I-W*. Given a parameter *α*, establishing the diffusion rate, and a preference vector *p_0_*, expressing the initial relevance score of each node, the ranking *p_α_* is given by
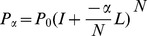
(9)where *N* is the number of iterations.

### Evaluation scheme

Leave-one-out cross-validation was conducted to evaluate performance of four ranking algorithms based on four networks. Then, based on the sensitivity and 1-specificity, ROC curves were drawn. In order to find out optimal performance of HKDR, PRW and SKM, a set of different parameters were empirically selected: HKDR with n = 2, 3, 4, 5, 6, 7; PRW with β = 0.01, 0.05, 0.1, 0.2, 0.3, 0.4,0.5,0.6, 0.7,0.8, 0.9,0.95; SKM with K = 2, 3, 4, 5, 6, 7, 8, 9.

## Supporting Information

Figure S1 **Measurement of the contributions of diverse datasets for constructing the FLN.** (A) GO semantic similarity. (B) Four microarray experiment datasets GSE1133, GSE4330, GSE6210, GSE4726. (C) Protein expression profiles. (D) Protein-protein interaction (PPI), Rosetta Stone (RS), domain-domain interaction (DDI), diseases involvements (DI), genetic interaction(GI). (E) Operons. (F) Phylogenetic profiles. (G) Phenotypic semantic similarity. (H) Shared domains.
(EPS)

Table S1 Ten-fold cross-validation results of machine-learning classifiers in Weka.
(DOC)

Table S2 List of high-confidence mammalian mitochondrial proteins.
(XLS)

Table S3 Coverage of datasets on gold standard set.
(DOC)

Table S4 Pearson correlation coefficients between high coverage datasets on the gold standard set.
(DOC)

Table S5 Optimal parameters and corresponding performances of four algorithms on four networks.
(DOC)

Table S6 Organisms used for phylogenetic profiles.
(XLS)

Method S1 **Data source and processing methods of the 11 genomic features to generate FLN.**
(DOC)
