# Supplementary material for: MitProNet: A Knowledgebase and Analysis Platform of Proteome, Interactome and Diseases for Mammalian Mitochondria
Source: PLoS One. 2014 Oct 27;9(10):e111187. doi: 10.1371/journal.pone.0111187 (PMC4210245; doi:10.1371/journal.pone.0111187)
Supplement: Table S1 — Ten-fold cross-validation results of machine-learning classifiers in Weka. (DOC) [file pone.0111187.s002.doc]

Table S1. Ten-fold cross-validation results of machine-learning classifiers in Weka

| **Model** | **TP** | **TPR** | **AUC** |
| --- | --- | --- | --- |
| AdaBoostM1 | 515 | 0.931 | 0.983 |
| Id3 | 486 | 0.879 | 0.94 |
| J48 | 515 | 0.931 | 0.959 |
| Logistic | 473 | 0.855 | 0.981 |
| MultiClassClassifier | 473 | 0.855 | 0.981 |
| MultilayerPerceptron | 471 | 0.852 | 0.976 |
| NaiveBayes | 483 | 0.873 | 0.984 |
| RandomForest | 476 | 0.861 | 0.967 |
