# Supplementary material for: MitProNet: A Knowledgebase and Analysis Platform of Proteome, Interactome and Diseases for Mammalian Mitochondria
Source: PLoS One. 2014 Oct 27;9(10):e111187. doi: 10.1371/journal.pone.0111187 (PMC4210245; doi:10.1371/journal.pone.0111187)
Supplement: Table S3 — Coverage of datasets on gold standard set. (DOC) [file pone.0111187.s004.doc]

**Table S3.** **Coverage of datasets on gold standard set.**

| **Datasets** | **Coverage of GSP** | **Coverage of GSN** |
| --- | --- | --- |
| GO Semantic Similarity | 0.792535 | 0.878763251 |
| GSE1133 | 0.6185 | 0.712879859 |
| GSE4330 | 0.598931 | 0.666696113 |
| GSE6210 | 0.656822 | 0.713038869 |
| GSE4726 | 0.501721 | 0.534946996 |
| Proteomics Profiles | 0.196775 | 0.207190813 |
| Phylogenetic Profiles | 0.99094 | 0.995689046 |
| Genetic Interaction | 0.00308 | 0.000106007 |
| Phenotypic semantic Similarity | 0.019569 | 0.022579505 |
| Diseases involvement | 0.005436 | 0.000335689 |
| Rosetta Stone | 0.004439 | 0.000812721 |
| Protein-protein Interaction | 0.023555 | 0.004717314 |
| Domain-domain Interaction | 0.034517 | 0.003109541 |
| Operon | 0.036148 | 0.017508834 |
| Shared Domains | 0.002808 | 0.000759717 |
