# Supplementary material for: MitProNet: A Knowledgebase and Analysis Platform of Proteome, Interactome and Diseases for Mammalian Mitochondria
Source: PLoS One. 2014 Oct 27;9(10):e111187. doi: 10.1371/journal.pone.0111187 (PMC4210245; doi:10.1371/journal.pone.0111187)
Supplement: Table S4 — Pearson correlation coefficients between high coverage datasets on the gold standard set. (DOC) [file pone.0111187.s005.doc]

**Table S4. Pearson correlation coefficients between high coverage datasets on the gold standard set.**

|  | **GO Semantic Similarity** | **GSE1133** | **GSE4330** | **GSE6210** | **GSE4726** | **Proteomics Profiles** | **Phylogenetic Profiles** |
| --- | --- | --- | --- | --- | --- | --- | --- |
| **GO Semantic Similarity** | 1 |  |  |  |  |  |  |
| **GSE1133** | 0.142413 | 1 |  |  |  |  |  |
| **GSE4330** | 0.063505 | 0.148045 | 1 |  |  |  |  |
| **GSE6210** | 0.016147 | 0.012315 | 0.053252 | 1 |  |  |  |
| **GSE4726** | 0.060626 | 0.12314 | 0.217074 | 0.185934 | 1 |  |  |
| **Proteomics Profiles** | 0.053059 | 0.055807 | 0.062298 | 0.090304 | 0.081638 | 1 |  |
| **Phylogenetic Profiles** | 0.123973 | 0.097611 | 0.065503 | 0.050071 | 0.091225 | 0.058239 | 1 |
