# Supplementary material for: MitProNet: A Knowledgebase and Analysis Platform of Proteome, Interactome and Diseases for Mammalian Mitochondria
Source: PLoS One. 2014 Oct 27;9(10):e111187. doi: 10.1371/journal.pone.0111187 (PMC4210245; doi:10.1371/journal.pone.0111187)
Supplement: Table S5 — Optimal parameters and corresponding performances of four algorithms on four networks. (DOC) [file pone.0111187.s006.doc]

**Table S5. Optimal parameters and corresponding performances of four algorithms on four networks**.

| **Methods** | **FLN** | | **FLNhm** | | **PPI network** | | **Co-expression network** | |
| --- | --- | --- | --- | --- | --- | --- | --- | --- |
|  | Parameters | AUC | Parameters | AUC | Parameters | AUC | Parameters | AUC |
| HKDR | N=3 | 0.880 | N=6 | 0.762 | N=4 | 0.748 | N=4 | 0.558 |
| PRP | *β*=0.95 | 0.870 | *β*=0.9 | 0.764 | *β*=0.9 | 0.748 | *β*=0.95 | 0.558 |
| KSM | K=7 | 0.836 | K=8 | 0.762 | K=3 | 0.767 | K=4 | 0.579 |
| AAR |  | 0.850 |  | 0.705 |  | 0.693 |  | 0.566 |

N: the number of iterations in HKDR algorithm; *β*: the back probability in PRP algorithm; K: the number of probability transition steps for KSM algorithm.
