# Supplementary material for: MitProNet: A Knowledgebase and Analysis Platform of Proteome, Interactome and Diseases for Mammalian Mitochondria
Source: PLoS One. 2014 Oct 27;9(10):e111187. doi: 10.1371/journal.pone.0111187 (PMC4210245; doi:10.1371/journal.pone.0111187)
Supplement: Method S1 — Data source and processing methods of the 11 genomic features to generate FLN. (DOC) [file pone.0111187.s008.doc]

**Method S1**

**Data source and processing methods of the 11 genomic features to generate FLN**

***Protein-protein interaction*:** Protein-protein interaction data were downloaded from HPRD database and I2d database. A binary score was used to denote the absence or presence of an interaction.

***Domain-domain interaction*:** Domain-domain interaction data was downloaded from 3did database. A binary score was used to denote whether the two proteins contain interacting domains.

***Shared domains*:** Domain data was downloaded from Interpro database. Each gene pair was scored by the number of domains encoded by both genes in the gene pair. The scores were divided into 3 bins.

***Genomic context*****:** As in genomic context, Rosetta Stone and phylogenetic profiles were selected as a genomic feature to construct FLN. Rosetta Stone data was downloaded from Prolinks. For each gene pairs, a score was assigned to denote whether the gene pair appears in a gene fusion event in other organism. The protein sequences of 600 organisms (Table S5) were downloaded from NCBI and KEGG. Then a blastp search between the 1086 mitochondiral genes and the 600 organisms proteins was performed, and the value 1 was assigned to indicate specific organism exists homology of the mitochondrial gene. Mutual information was used to measure correlation of gene pairs based on phylogenetic profiles data, which was calculated by mutualInfo function in bioDist, a R package . The mutual information has values between 0 and 1, and we divided them into six bins.

***GO*** ***semantic similarity*:** GO data was retrieved from the GO database. Correlation of genes sharing the same biological process terms was calculated by Rgui package GOSemSim . The GO semantic similarity has values between 0 and 1, and we divided them into six bins.

***Genetic interaction*:** Gene interactions between yeast genes were downloaded from SGD database. A binary scorewas used to denote the absence or presence of genetic interaction of a gene pair.

***Phenotypic sementic similarity*:** Mouse phenotypic data was retrieved from Mammalian Phenotype Browser. A method in terms of Smallest Shared Mammalian Phenotypes was used to measure the correlation of gene pairs sharing the similar phenotypic data. Smallest Shared Mammalian Phenotypes is based on the premise that a pair of proteins may be highly associated in their functions if they share a small and specific phenotypic annotation and were calculated by three steps: (1) search all the phenotypic terms shared by the pair of proteins, (2) for each of the terms found in step 1, find the number of other proteins also sharing this term, (3) get the phenotypic term with the smallest number of proteins annotated with the term, get the protein count. The protein counts were further divided to five bins.

***Co-expression*:** Four microarray experiments datasets, GSE1133 , GSE4330, GSE6210 and GSE4726 were included as a genomic feature to identify gene pairs with associated functions. The datasets of the four microarray experiments were downloaded from gene expression omnibus (GEO) database. Pearson correlation coefficient (PCC) of gene pairs was calculated to identify gene pairs with related functions. The PCC has values between -1 and 1, and we divided them into 20 bins with an interval of 0.1.

***Protein expression profiles*:** We used the results of Thomas Kislinger et al’s proteomics experiments to build the protein expression profiles and then calculated the spearman correlation coefficient of gene pairs which encode the proteins in the protein expression profiles. The spearman correlation coefficient has values between -1 and 1, and we divided them into 20 bins with an interval of 0.1, similar with PCC.

***Shared diseases*:** Disease data was retrieved from OMIM database. We used binary score to denote whether the gene pairs exist in the same diseases in OMIM.

***Operon*:** We retrieved operon data from Database of prOkaryotic OpeRons. Each gene pair was then scored by the number of operons containing both genes. The scores were divided into 3 bins.

1. Ding B, Gentleman R, Carey V (2010) bioDist: Different distance measures. R package version 1.

2. Yu G, Li F, Qin Y, Bo X, Wu Y, et al. (2010) GOSemSim: an R package for measuring semantic similarity among GO terms and gene products. Bioinformatics 26: 976-978.

3. Su AI, Wiltshire T, Batalov S, Lapp H, Ching KA, et al. (2004) A gene atlas of the mouse and human protein-encoding transcriptomes. Proceedings of the National Academy of Sciences of the United States of America 101: 6062-6067.

4. Calvo S, Jain M, Xie X, Sheth SA, Chang B, et al. (2006) Systematic identification of human mitochondrial disease genes through integrative genomics. Nature genetics 38: 576-582.

5. Vianna CR, Huntgeburth M, Coppari R, Choi CS, Lin J, et al. (2006) Hypomorphic mutation of PGC-1β causes mitochondrial dysfunction and liver insulin resistance. Cell metabolism 4: 453-464.

6. Siddiqui AS, Khattra J, Delaney AD, Zhao Y, Astell C, et al. (2005) A mouse atlas of gene expression: large-scale digital gene-expression profiles from precisely defined developing C57BL/6J mouse tissues and cells. Proceedings of the National Academy of Sciences of the United States of America 102: 18485-18490.
